# Rates and causes of death after release from incarceration: an individual participant data meta-analysis from 1,471,526 people in eight high- and middle-income countries

**DOI:** 10.23889/ijpds.v9i5.2489

**Published:** 2024-09-18

**Authors:** Rohan Borschmann, Stuart Kinner, MARIC team

**Affiliations:** 1 University of Melbourne; 2 University of Oxford; 3 Curtin University

## Background

Formerly incarcerated people have exceptionally poor health profiles and are at increased risk of preventable mortality when compared to their general population peers. However, not enough is known about the epidemiology of mortality in this population – specifically the rates, causes, and timing in specific subgroups and regions – to inform the development of targeted, evidence-based responses.

## Methods

We examined mortality outcomes for 1,471,526 people released from incarceration in eight countries from 1980-2018, across 10,534,441 person-years of follow-up (range: 0-24 years per person). We combined data from 18 cohort studies using two-step individual participant data meta-analyses to estimate pooled all-cause and cause-specific crude mortality rates (CMRs) per 100,000 person-years, with 95% confidence intervals (95%CI), for specific time periods after release, overall and stratified by age, sex, and region.

## Findings

75,427 deaths were recorded. The all-cause CMR was highest during the first week. The highest cause-specific rates of death during the first week were due to alcohol and other drug poisoning, suicide, and cardiovascular disease. We observed considerable variation in cause-specific CMRs over time since release and across regions. Pooled all-cause CMRs were similar between males and females, and were higher in older age groups.

## Interpretation

The markedly elevated rate of death in the first week post-release underscores an urgent need for investment in evidence-based, coordinated transitional healthcare, including treatment for mental illness and substance use disorders. Variations in both rates and causes of death over time highlight the need for routine monitoring of post-release mortality.

